# Exploring Serum Anti-thyroid Peroxidase Antibodies and High-Sensitivity C-reactive Protein as Inflammatory Markers in Subclinical Hypothyroidism: A Comprehensive Study

**DOI:** 10.7759/cureus.76906

**Published:** 2025-01-04

**Authors:** Anantha Priya, Barani Sri, Ganesh Bala, Suganya Kannan

**Affiliations:** 1 Biochemistry, Vinayaka Mission's Medical College, Vinayaka Mission’s Research Foundation (Deemed to be University) - Karaikal, Puducherry, IND; 2 ENT, Vinayaka Mission's Medical College, Vinayaka Mission’s Research Foundation (Deemed to be University) - Karaikal, Puducherry, IND; 3 Central Research Laboratory, Vinayaka Mission's Medical College, Vinayaka Mission’s Research Foundation (Deemed to be University) - Karaikal, Puducherry, IND

**Keywords:** anti-thyroid peroxidase antibodies (anti-tpo), autoimmune thyroiditis, high-sensitive c-reactive protein (hscrp), inflammatory markers, subclinical hypothyroidism

## Abstract

Background and aim

Subclinical hypothyroidism (SCH) is a biochemical condition defined by elevated thyroid-stimulating hormone (TSH) levels with normal free thyroxine (FT4) levels, often occurring without overt clinical symptoms. Emerging evidence suggests a role for autoimmune and inflammatory processes, with anti-thyroid peroxidase antibodies (anti-TPO) and high-sensitivity C-reactive protein (hsCRP) as potential markers of thyroid autoimmunity and systemic inflammation. This study evaluates the significance of these markers in SCH to elucidate their association with disease progression and clinical symptoms. This study aims to assess the roles of anti-TPO and hsCRP as inflammatory markers in SCH and to examine their correlations with clinical symptoms, lipid profiles, and cardiovascular risk, to stratify patients based on their autoimmune and inflammatory profiles.

Methodology

A retrospective, cross-sectional study was conducted on 220 patients, including 170 diagnosed with SCH and 50 euthyroid controls. Patients were categorized into SCH with anti-TPO positivity (SCH+), SCH without anti-TPO positivity (SCH-), and euthyroid controls. Serum TSH, FT4, anti-TPO, and hsCRP levels were analyzed using chemiluminescent immunoassay (CLIA), enzyme-linked immunosorbent assay (ELISA), and turbidimetric immunoassay. Statistical analyses, including Kruskal-Wallis and correlation tests, evaluated associations between hsCRP, anti-TPO, and clinical parameters.

Results

SCH+ participants demonstrated significantly higher levels of TSH (7.6 ± 2.1 mIU/L), hsCRP (4.2 ± 1.6 mg/L), and anti-TPO antibodies (116.5 ± 34.8 IU/mL) compared to SCH- and controls (*P* < 0.001). hsCRP was elevated in 52.2% of SCH+ individuals, indicating a systemic inflammatory state, compared to 32.1% in SCH- and 16% in controls. Significant correlations were observed between hsCRP and TSH (*r* = 0.62, *P* < 0.001) and between hsCRP and anti-TPO (*r* = 0.58, *P* < 0.001) in SCH+ participants. SCH+ patients exhibited a higher prevalence of fatigue, cold intolerance, and lipid abnormalities, with total cholesterol and LDL levels markedly elevated compared to other groups.

Conclusions

Anti-TPO and hsCRP are valuable markers for identifying systemic inflammation and autoimmune activity in SCH, particularly in anti-TPO-positive individuals. Their integration into routine evaluation may help stratify SCH patients based on cardiovascular and metabolic risk, guiding early therapeutic interventions. Further, longitudinal studies are needed to explore their prognostic value and therapeutic implications.

## Introduction

Subclinical hypothyroidism (SCH) is a biochemical condition characterized by elevated serum thyroid-stimulating hormone (TSH) levels with normal free thyroxine (FT4) levels, often in the absence of overt clinical symptoms of hypothyroidism. While frequently detected during routine laboratory evaluations, particularly in middle-aged and elderly individuals, SCH has traditionally been regarded as a benign condition or a precursor to overt hypothyroidism. However, emerging evidence suggests that SCH may have broader implications, including systemic metabolic, cardiovascular, and inflammatory consequences [1]. Understanding the pathophysiological underpinnings of SCH is critical for determining appropriate treatment thresholds and mitigating its associated risks.

Autoimmune thyroiditis, most notably Hashimoto’s thyroiditis, is a common cause of SCH, particularly in iodine-sufficient populations. This autoimmune condition involves the progressive destruction of thyroid tissue through immune-mediated mechanisms, primarily driven by autoantibodies such as anti-thyroid peroxidase antibodies (anti-TPO). Elevated anti-TPO levels serve as a hallmark of autoimmune thyroid disorders and indicate an ongoing inflammatory process within the thyroid gland [2]. Notably, patients with SCH and high anti-TPO antibody titers are at greater risk of progressing to overt hypothyroidism. Despite these associations, the role of anti-TPO as a diagnostic and prognostic marker in SCH requires further elucidation, particularly regarding its contribution to systemic inflammatory processes.

Systemic inflammation has also been implicated in SCH, with high-sensitivity C-reactive protein (hsCRP) emerging as a valuable marker of chronic low-grade inflammation. Elevated hsCRP levels have been associated with conditions such as cardiovascular disease (CVD) and metabolic syndrome, raising questions about the potential systemic consequences of SCH. Several studies have reported correlations between SCH and elevated hsCRP levels, suggesting that SCH may contribute to or exacerbate a pro-inflammatory state [3]. This intersection of thyroid dysfunction, autoimmunity, and systemic inflammation warrants deeper exploration, as it could reveal novel pathways linking SCH to adverse metabolic and cardiovascular outcomes.

Despite the growing recognition of inflammation as a key component of SCH pathophysiology, existing research has often considered autoimmune mechanisms (anti-TPO) and systemic inflammatory markers (hsCRP) in isolation. Integrating these markers may provide a more comprehensive understanding of SCH and its clinical implications [4]. Anti-TPO reflects localized thyroid autoimmunity, while hsCRP provides insight into systemic inflammation that may extend beyond the thyroid gland. Concurrent evaluation of these markers could enhance diagnostic and prognostic precision, particularly in identifying individuals at heightened risk for disease progression or complications [5].

The current study addresses these knowledge gaps by investigating the roles of anti-TPO and hsCRP as inflammatory markers in SCH. Specifically, we aim to (1) evaluate the prevalence and significance of elevated anti-TPO and hsCRP levels in SCH compared to other thyroid disorder subgroups and healthy controls; (2) examine the relationship between anti-TPO and hsCRP levels and their potential interactions in driving systemic inflammation; and (3) assess the association between elevated levels of these markers and the risk of progression to overt hypothyroidism, CVD, or metabolic complications. By integrating these objectives, this study seeks to identify patients who may benefit from early interventions, offering new insights into the intersection of thyroid autoimmunity and systemic inflammation in SCH.

The findings of this study could have significant clinical implications, particularly in stratifying SCH patients based on their inflammatory and autoimmune profiles, guiding therapeutic decisions, and identifying individuals at risk for adverse outcomes. This timely research bridges the gap in understanding how autoimmune and systemic inflammatory processes intersect in the context of SCH, offering a more holistic perspective on its pathophysiology and progression.

## Materials and methods

Study design

This study was designed as a cross-sectional, observational study, conducted to assess the roles of serum anti-TPO and hsCRP as inflammatory markers in patients with SCH. The data were collected retrospectively from patient records over a defined time frame, as described next.

Ethical approval

Ethical approval for this study was obtained from the Institutional Review Board or Ethics Committee at Vinayaka Mission’s Medical College and Hospital, in accordance with the principles outlined in the Declaration of Helsinki. All participants provided informed consent for the use of their clinical data in research. The study protocol was reviewed and approved, ensuring compliance with all applicable ethical standards for research involving human subjects.

Study population

The study examined data from 220 patients diagnosed with various thyroid disorders, including 106 men and 114 women, aged 18 to 72 years (mean 56.2 ± 5.0 years; median 52 years). These patients were treated or diagnosed in both outpatient (140 patients) and inpatient (80 patients).

Participant recruitment

This study was conducted as a retrospective, single-center, cross-sectional analysis of patients diagnosed with thyroid disorders at Vinayaka Mission’s Medical College and Hospital between January 2021 and December 2023. The source of data included clinical and laboratory records of patients treated or diagnosed in both outpatient and inpatient settings. A total of 220 participants were included in the study, comprising 106 men and 114 women aged 18-72 years.

Recruitment process

Patients were recruited through routine clinical care pathways. The study population consisted of 140 outpatients attending thyroid disorder clinics and 80 inpatients who were admitted to the hospital for further evaluation or management of thyroid-related or general medical conditions.

Patients were selected based on laboratory-confirmed thyroid dysfunction as determined by TSH and FT4 levels. Recruitment was performed by trained clinical staff, and inclusion and exclusion criteria were strictly applied to ensure homogeneity of the study population. Randomized selection was employed to ensure an unbiased representation of participants across all thyroid dysfunction subgroups.

Patient selection and exclusion criteria

Inclusion criteria required patients to have a confirmed diagnosis of thyroid dysfunction, including SCH, hyperthyroidism, or euthyroid states, based on laboratory results. Exclusion criteria included patients with concurrent injuries, rheumatic diseases, dental disease, cancer (excluding thyroid cancer), and those who smoked cigarettes. Postmenopausal women receiving hormonal replacement therapy were included to account for this patient group in the analysis.

Clinical parameters

Descriptive analysis was conducted to outline the distribution of the study group within the study population. To ensure uniformity and accuracy in biochemical measurements, all samples were collected in the morning between 8:00 AM and 10:00 AM to minimize diurnal variations in thyroid hormone and hsCRP levels. Participants were instructed to fast for 8-12 hours before sample collection, eliminating potential postprandial effects on biochemical parameters such as hsCRP and lipid profile. This fasting protocol was consistently applied across all study groups, including the SCH+ group, SCH- group, and the euthyroid control group, to maintain comparability of results. Categorical variables, such as the presence of weakness, lethargy, swellings, intolerance to cold, constipation, weight gain, coarse dry skin, hair fall, menorrhagia, infertility, mood changes, and depression, were compared between the case and control groups.

Serum samples were separated within two hours of collection by centrifugation at 3,000 rpm for 10 minutes and stored at -20 °C until analysis. Thyroid function tests, including TSH and FT4, were measured using a chemiluminescent immunoassay (CLIA) on an automated analyzer (Abbott Architect i1000SR, Abbott Diagnostics, Chicago, IL), with reference ranges of 0.4-4.5 mIU/L for TSH and 0.8-1.8 ng/dL for FT4. Anti-TPO antibody levels were assessed using the Monobind AccuBind ELISA kit (Monobind Inc., USA), with a cutoff of 34 IU/mL. hsCRP levels were determined using the Erba XL-200 Turbidimetric Immunoassay kit (Erba Mannheim, India), with a detection limit of 0.1 mg/L and a normal range of ≤3 mg/L. Quality control measures ensured precision, with intra- and inter-assay coefficients of variation maintained below 5%. All laboratory analyses were conducted in the hospital’s clinical biochemistry laboratory, adhering to standardized protocols and manufacturer guidelines. Data were meticulously reviewed for accuracy and completeness before inclusion in the study.

Patient subgroups

The study population was categorized into three distinct subgroups to facilitate a comparative analysis of inflammatory markers and clinical profiles across different thyroid states.

The first subgroup consisted of patients with SCH accompanied by anti-TPO antibody positivity (SCH+). These individuals had elevated TSH levels and normal FT4 levels, with the presence of anti-TPO antibodies indicating an autoimmune basis for their thyroid dysfunction. This subgroup was characterized by a heightened inflammatory profile, reflecting the interplay between thyroid autoimmunity and systemic inflammation.

The second subgroup included patients with SCH who were anti-TPO negative (SCH-). While these individuals also exhibited elevated TSH and normal FT4 levels, the absence of anti-TPO antibodies suggested non-autoimmune mechanisms underlying their thyroid dysfunction. This subgroup provided a contrast to the SCH+ group, allowing for the assessment of inflammation in the absence of thyroid autoimmunity.

The third subgroup comprised euthyroid individuals who served as the control group. These participants had normal TSH and FT4 levels and no detectable anti-TPO antibodies. The control group provided a baseline for evaluating the extent of inflammation and clinical symptoms associated with thyroid dysfunction.

Categorization of study and control groups

The study group consisted of 170 patients diagnosed with SCH, defined by elevated TSH levels (>4.5 mIU/L) and normal FT4 levels, and further categorized into two subgroups: SCH+ (SCH with anti-TPO positivity), comprising patients with detectable anti-TPO antibodies, indicating an autoimmune etiology; and SCH- (SCH without anti-TPO positivity), consisting of patients with no detectable anti-TPO antibodies, suggesting a non-autoimmune cause of thyroid dysfunction. The control group included 50 euthyroid individuals with normal TSH and FT4 levels and no detectable anti-TPO antibodies, serving as a reference population to compare inflammatory markers, biochemical parameters, and clinical profiles against the SCH groups.

Diagnostic criteria

Elevated free thyroid hormones and suppressed thyrotropin (TSH) were the diagnostic criteria for hyperthyroidism. The criteria for subclinical hyperthyroidism were normal free thyroid hormones and suppressed TSH. The diagnosis of hypothyroidism was established on the premise of elevated TSH and decreased free thyroid hormones.

Experimental Protocol

Most participants were hyperthyroid patients preparing for radioiodine therapy, while others were recruited from outpatient clinics. hsCRP levels were measured to assess the inflammatory process.

Laboratory Methods

Various laboratory tests were conducted, including the measurement of free T4, free T3, TSH, thyroglobulin antibodies (Tg-Abs), TPO antibodies (TPO-Abs), TSH receptor antibodies (TSHR-Abs), hsCRP, anti-TPO, and lipid profile parameters (total cholesterol [TC], triglycerides [TG], high-density lipoprotein [HDL], low-density lipoprotein [LDL], and very low-density lipoprotein [VLDL]). Reference ranges for each parameter were established based on standard values.

Treatment

Treatment strategies varied based on the specific thyroid disorder, ranging from beta-blockers for symptomatic relief in hyperthyroidism to antithyroid drugs, radioiodine therapy, and hormone replacement therapy for hypothyroidism.

Statistical analysis

Statistical 10 from StatSoft was employed to conduct the statistical analyses. The Kruskal-Wallis test was implemented as a result of the distribution of hsCRP values in the individual groups being non-normal. To evaluate the impact of hsCRP on autoantibodies (Tg-Abs, TPO-Abs, and TSHR-Abs) and TSH, correlation analyses were implemented, including Pearson linear correlation and Spearman's rank correlation, with the normality of variable distributions in mind. The significance level was established at *P* < 0.05.

## Results

Our study aimed to investigate the roles of serum anti-TPO and hsCRP as inflammatory markers in SCH. The results are presented in several sections, including descriptive analysis, comparison of categorical variables, mean value comparisons, autoantibody and TSH receptor antibody comparisons, correlation analyses, and subgroup analyses.

Descriptive analysis of the study population

A total of 220 participants were included in the study, comprising 106 men and 114 women, aged 18 to 72 years (mean age: 56.2 ± 5.0 years; median: 52 years). Of these, 140 patients were recruited from outpatient settings, and 80 were admitted through inpatient care at Vinayaka Mission’s Medical College. Among the women, 76 (67%) were diagnosed with SCH, while among the men, 35 (33%) were diagnosed with SCH. This resulted in a total of 111 participants diagnosed with SCH across both genders. The study population was further categorized into three groups: 92 participants (42%) had SCH with anti-TPO positivity (SCH+), 79 participants (36%) had SCH without anti-TPO positivity (SCH−), and 49 participants (22%) were euthyroid individuals, forming the control group.

SCH With Anti-TPO Positivity (SCH+)

This subgroup included 92 participants, representing individuals with elevated TSH levels and normal FT4 levels along with detectable anti-TPO antibodies. These findings indicated an autoimmune etiology for their thyroid dysfunction. SCH+ participants formed the largest subgroup with thyroid autoimmunity, characterized by a heightened inflammatory response and a higher prevalence of clinical symptoms such as fatigue, cold intolerance, and weight gain.

SCH without Anti-TPO Positivity (SCH-)

This subgroup comprised 78 participants who also had elevated TSH levels and normal FT4 levels but lacked detectable anti-TPO antibodies. SCH- individuals were likely to have non-autoimmune thyroid dysfunction and showed a lower inflammatory burden compared to the SCH+ subgroup, with fewer clinical symptoms.

Control Group (Euthyroid)

The control group consisted of 50 participants with normal TSH and FT4 levels and no detectable anti-TPO antibodies. This group served as the baseline for evaluating the inflammatory markers and clinical manifestations in the SCH+ and SCH- subgroups. Euthyroid individuals displayed minimal symptoms and no significant elevations in inflammatory markers like hsCRP.

The mean TSH levels were significantly higher in the SCH+ group (7.6 ± 2.1 mIU/L) compared to the SCH- group (5.8 ± 1.9 mIU/L) and the control group (2.1 ± 0.8 mIU/L). Additionally, elevated serum hsCRP levels were observed in 48% of SCH+ participants, indicating a potential inflammatory state associated with SCH.

Comparison of categorical variables

A comparison of categorical variables revealed significant differences in the prevalence of clinical symptoms across the study groups. Fatigue and weakness were reported in 85% of SCH+ participants compared to 58% of SCH- participants and 22% of the control group, with a statistically significant difference (*P* < 0.001). Cold intolerance, a hallmark symptom of hypothyroidism, was present in 76% of SCH+ participants, 52% of SCH-, and 18% of controls (*P* < 0.001). Other notable symptoms, including constipation, weight gain, and mood changes, were also more prevalent in SCH+ individuals, underscoring a distinct clinical profile associated with anti-TPO positivity.

Comparison of mean values

The mean values of TSH, FT4, hsCRP, and BMI were compared across the groups. TSH levels were significantly higher in the SCH+ group compared to the SCH- group and controls (*P* < 0.001). Serum hsCRP levels were markedly elevated in SCH+ participants (4.2 ± 1.6 mg/L) compared to SCH- (3.1 ± 1.2 mg/L) and controls (2.0 ± 0.9 mg/L), indicating an inflammatory trend associated with anti-TPO positivity (*P* < 0.001). There was no significant difference in FT4 levels between SCH+ and SCH- participants, suggesting that FT4 remained stable in SCH. BMI was slightly elevated in both SCH groups compared to controls, with a mean BMI of 28.4 ± 3.5 kg/m² in SCH+ participants.

Autoantibodies and TSHR Abs

Serum anti-TPO antibody levels were significantly higher in SCH+ participants (mean: 116.5 ± 34.8 IU/mL) compared to SCH- participants (mean: 22.1 ± 5.6 IU/mL) and controls (mean: 18.5 ± 3.2 IU/mL) (*P* < 0.001). Anti-TPO positivity was associated with higher TSH levels and elevated hsCRP, suggesting a link between autoimmunity and systemic inflammation. TSH receptor antibody levels were measured in a subset of participants, but no significant differences were observed between SCH+ and SCH- groups.

Correlation analysis

Correlation analysis demonstrated a positive correlation between serum TSH levels and hsCRP concentrations (*r* = 0.62, *P* < 0.001). Anti-TPO antibody levels were also positively correlated with hsCRP levels (*r* = 0.58, *P* < 0.001), indicating a strong association between thyroid autoimmunity and systemic inflammation. No significant correlation was observed between FT4 and hsCRP levels, further highlighting the role of TSH and autoantibodies as inflammatory mediators in SCH.

Subgroup analysis

Subgroup analysis revealed distinct inflammatory patterns within SCH+ and SCH- participants. Elevated hsCRP levels were observed in 54% of SCH+ participants compared to 32% of SCH- participants, suggesting that anti-TPO positivity is a key driver of inflammation in SCH (*P* < 0.001). Among patients with elevated hsCRP, fatigue, and cold intolerance were more frequently reported in SCH+ individuals compared to their SCH- counterparts. In the control group, hsCRP levels remained within normal limits, and clinical symptoms were less frequent.

This analysis confirms the role of serum anti-TPO and hsCRP as potential inflammatory markers in SCH, with a stronger inflammatory profile observed in patients with anti-TPO positivity (Tables 1-8).

**Table 1:** Descriptive analysis of the study group in the study population. hsCRP, high-sensitivity C-reactive protein; TPO, thyroid peroxidase antibodies; SCH, subclinical hypothyroidism

Parameter	SCH+ (*n *= 92)	SCH- (*n *= 78)	Controls (*n *= 50)	Total (*n* = 220)
Gender (Male/Female)	32/60	28/50	46/54	106/114
Mean age (years)	57.2 ± 6.1	55.3 ± 5.4	53.8 ± 4.8	56.2 ± 5.0
Elevated hsCRP (%)	48 (52.2%)	25 (32.1%)	8 (16%)	81 (36.8%)
Positive anti-TPO (%)	92 (100%)	0 (0%)	0 (0%)	92 (41.8%)
TSH > 4.5 mIU/L (%)	92 (100%)	78 (100%)	0 (0%)	170 (77.3%)

**Table 2:** Descriptive analysis of the study group in the study population.

Symptom	SCH+ (*n *= 92)	SCH- (*n *= 78)	Controls (*n *= 50)	*P*-value
Fatigue, *n* (%)	78 (84.8%)	45 (57.7%)	12 (24%)	<0.001
Cold intolerance, *n* (%)	70 (76%)	41 (52.6%)	9 (18%)	<0.001
Constipation, *n* (%)	60 (65.2%)	38 (48.7%)	7 (14%)	<0.001
Depression, *n* (%)	55 (59.8%)	30 (38.5%)	5 (10%)	<0.001

**Table 3:** Comparison of mean values between case and control groups. TSH, thyroid-stimulating hormone; hsCRP, high-sensitivity C-reactive protein; BMI, body mass index

Parameter	SCH+ (*n* = 92)	SCH- (*n* = 78)	Controls (*n* = 50)	*P*-value
TSH (mIU/L)	7.6 ± 2.1	5.8 ± 1.9	2.1 ± 0.8	<0.001
hsCRP (mg/L)	4.2 ± 1.6	3.1 ± 1.2	2.0 ± 0.9	<0.001
BMI (kg/m²)	28.4 ± 3.5	27.5 ± 3.2	25.2 ± 2.8	<0.001

**Table 4:** Comparison of autoantibodies and TSH receptor antibodies between case and control groups. TSH, thyroid-stimulating hormone; TPO, thyroid peroxidase antibodies; SCH, subclinical hypothyroidism

Autoantibody	SCH+ (*n* = 92)	SCH- (*n* = 78)	Controls (*n* = 50)	*P*-value
Anti-TPO (IU/mL)	116.5 ± 34.8	22.1 ± 5.6	18.5 ± 3.2	<0.001
TSH receptor antibodies	0.4 ± 0.2	0.3 ± 0.1	0.3 ± 0.1	0.07

**Table 5:** Correlation analysis between hsCRP and other parameters in the study group. TSH, thyroid-stimulating hormone; hsCRP, high-sensitivity C-reactive protein; BMI, body mass index; TPO, thyroid peroxidase antibodies

Parameter	Correlation coefficient (*r*)	*P*-value
TSH	0.62	<0.001
Anti-TPO	0.58	<0.001
BMI	0.41	<0.01

**Table 6:** Subgroup analysis of hsCRP levels within each thyroid disorder subgroup. hsCRP, high-sensitivity C-reactive protein; SCH, subclinical hypothyroidism

Group	Elevated hsCRP (*n*)	Normal hsCRP (*n*)	Percentage elevated (%)
SCH+	48	44	52.2
SCH-	25	53	32.1
Controls	8	42	16.0

**Table 7:** Subgroup analysis of lipid profile parameters within each thyroid disorder subgroup. SCH, subclinical hypothyroidism; LDL, low-density lipoprotein; HDL, high-density lipoprotein

Parameter	SCH+ (*n *= 92)	SCH- (*n *= 78)	Controls (*n *= 50)	*P*-value
Total cholesterol (mg/dL)	210 ± 36	190 ± 28	176 ± 22	<0.001
LDL (mg/dL)	135 ± 26	115 ± 20	102 ± 18	<0.001
HDL (mg/dL)	46 ± 7	50 ± 8	54 ± 9	<0.01
Triglycerides (mg/dL)	160 ± 30	140 ± 26	130 ± 24	<0.01

**Table 8:** Correlation analysis between hsCRP and other parameters in study and control groups. TSH, thyroid-stimulating hormone; hsCRP, high-sensitivity C-reactive protein; BMI, body mass index; TPO, thyroid peroxidase antibodies

Parameter	SCH+ (*n* = 92)	SCH- (*n* = 78)	Controls (*n *= 50)	*P*-value
TSH vs. hsCRP	*r* = 0.62	*r* = 0.35	No correlation	<0.001
Anti-TPO vs. hsCRP	*r* = 0.58	No correlation	No correlation	<0.001
BMI vs. hsCRP	*r* = 0.41	*r* = 0.30	No correlation	<0.01

## Discussion

This study investigated the roles of serum anti-TPO and hsCRP as potential inflammatory markers in SCH. By analyzing a cohort of 220 patients stratified by anti-TPO positivity, hsCRP levels, and thyroid function, we have provided detailed insights into the complex interplay between thyroid autoimmunity, systemic inflammation, and clinical symptoms. The findings are significant not only for advancing our understanding of SCH but also for highlighting the clinical implications of inflammatory markers in this condition.

Our results reveal that anti-TPO positivity, a hallmark of autoimmune thyroiditis, is associated with elevated hsCRP levels and more pronounced clinical symptoms in SCH+ patients compared to SCH- patients and controls. This finding underscores the inflammatory and autoimmune nature of SCH+ and its potential role as a driver of systemic inflammation. These observations align with established knowledge, such as the work by Pearce and Leech, who highlighted anti-TPO as a critical marker for autoimmune thyroiditis. However, our study uniquely emphasizes the relationship between anti-TPO and systemic inflammatory markers like hsCRP, providing evidence that thyroid autoimmunity has implications beyond the thyroid gland itself [6].

Elevated hsCRP levels, observed in 52.2% of SCH+ participants, compared to 32.1% in SCH- and 16% in controls, are indicative of a low-grade systemic inflammatory state. These findings align with prior studies, such as those by Duntas and Biondi, who suggested that inflammation in SCH may be a key mediator of its associated comorbidities, including CVDs [7]. Our study adds to this body of evidence by demonstrating that hsCRP elevation is significantly correlated with TSH and anti-TPO levels (*r* = 0.62 and *r* = 0.58, respectively), suggesting a direct link between thyroid dysfunction, autoimmunity, and inflammation. This correlation supports the hypothesis that elevated TSH, as seen in SCH, may contribute to oxidative stress and inflammatory responses, as proposed by Münzker et al. [8].

From a molecular perspective, the association between anti-TPO and inflammation can be explained by the immune-mediated destruction of thyroid tissue, which releases danger-associated molecular patterns (DAMPs) that activate toll-like receptors (TLRs). This activation triggers the production of pro-inflammatory cytokines such as interleukin-6 (IL-6), tumor necrosis factor-alpha (TNF-α), and CRP. de Araújo et al. postulated that such cytokine release not only sustains localized thyroid inflammation but also propagates systemic inflammatory responses, as evidenced by elevated hsCRP levels in our SCH+ participants. This immune-inflammatory feedback loop may explain the significant symptom burden, such as fatigue, cold intolerance, and depression, observed in SCH+ individuals compared to SCH- and controls [9].

The clinical symptoms reported by our participants further reinforce the inflammatory nature of SCH. SCH+ patients exhibited significantly higher rates of fatigue (84.8%), cold intolerance (76%), and depression (59.8%) compared to SCH- (57.7%, 52.6%, and 38.5%, respectively) and controls (24%, 18%, and 10%, respectively). These findings are consistent with the work of McAninch and Bianco, who highlighted that SCH, particularly when associated with autoimmunity, is characterized by symptoms linked to both thyroid hormone insufficiency and systemic inflammation [10]. Elevated hsCRP in SCH+ patients likely contributes to these symptoms, as inflammatory markers such as CRP have been implicated in neuropsychiatric and somatic symptomatology, as demonstrated by Thvilum et al. [11].

Lipid profile abnormalities in SCH+ participants were another notable finding of this study. Elevated total cholesterol (210 ± 36 mg/dL) and LDL cholesterol (135 ± 26 mg/dL) in SCH+ participants compared to SCH- and controls highlight the metabolic impact of thyroid dysfunction and autoimmunity. Dyslipidemia is a well-documented feature of SCH, as shown by Wu et al., but our study provides evidence that these abnormalities are more pronounced in anti-TPO-positive individuals. The interplay between inflammation and lipid metabolism may explain this finding, as hsCRP is not only a marker of inflammation but also a predictor of lipid metabolism disturbances. This dual role of hsCRP underscores its clinical utility in identifying SCH+ patients at higher cardiovascular risk [12].

The clinical implications of our findings are significant. First, the consistent elevation of hsCRP and anti-TPO levels in SCH+ participants suggests that these markers could be used to stratify patients based on their inflammatory and cardiovascular risk profiles. Routine assessment of hsCRP and anti-TPO in SCH patients could help identify individuals who may benefit from early interventions, such as lifestyle modifications, anti-inflammatory therapies, or even early initiation of levothyroxine therapy, as suggested by Biondi and Cooper [13]. Second, our findings highlight the need for a more nuanced approach to SCH management, recognizing the heterogeneity of this condition and the distinct inflammatory and clinical profiles of SCH+ and SCH- subgroups [13].

While our study provides valuable insights, it also raises important questions about the long-term impact of inflammation in SCH and the potential benefits of targeted interventions. For instance, could anti-inflammatory therapies mitigate hsCRP elevation and improve clinical outcomes in SCH+ patients? Could earlier treatment with levothyroxine prevent the progression of cardiovascular and metabolic complications in these individuals? Future longitudinal studies are needed to address these questions and to explore the causal relationships between thyroid autoimmunity, systemic inflammation, and clinical outcomes.

In conclusion, this study highlights the significant role of anti-TPO and hsCRP as inflammatory markers in SCH, particularly in anti-TPO-positive individuals. The findings emphasize the importance of thyroid autoimmunity in driving systemic inflammation and its associated clinical manifestations. By identifying SCH+ patients with elevated hsCRP and anti-TPO, clinicians can better stratify risk and tailor interventions, ultimately improving patient outcomes. These insights pave the way for a more personalized approach to the management of SCH, addressing not only thyroid function but also its systemic inflammatory consequences.

## Conclusions

Our comprehensive study demonstrates that serum anti-TPO and hsCRP are valuable inflammatory markers in subclinical hypothyroidism. The elevated levels of these markers highlight the significant autoimmune and inflammatory components of this condition. Understanding the roles of anti-TPO and hsCRP can refine diagnostic and therapeutic approaches, promoting personalized care for patients with subclinical hypothyroidism. Future research should continue to explore these relationships, aiming to improve outcomes for individuals affected by this common yet complex thyroid disorder.
